# Researches on Chronic Inflammation of the Cerebral Vessels

**Published:** 1829-04-01

**Authors:** 


LXII.

### HOTEL DIEU—COCHIN—LA PITIE.

Researches tending to prove that Chonic Inflammation of the Cerebral Vessels plays an important Part in the Production of Apoplexy. By M. Bouillaud, M.D.

To the difficulties thrown in the way of the medical practitioner, as well as of the physiologist and pathologist, apoplexy contributes its share. Few physicians, when called to a man in a fit of apoplexy, dare attempt to say whether the cause of the pressure on the brain be within or without the vessels—or whether the attack is likely to pass off or prove fatal.

It is the intention of our author, in the memoir now before us, to consider apoplexy and cerebral hæmorrhage as synonymous, and to endeavour to shew, by facts, that there is a considerable analogy between cerebral hæmorrhage and hæmorrhage in general, whether the blood be thrown out by exhalation or by rupture of vessels. The hæmorrhages by rupture may be caused by external violence or by internal lesion, such as ulceration resulting from chronic inflammation of the coats of vessels. It is to this cause that M. Bouillaud particularly directs his attention. The author has shewn, in a former memoir, that chronic inflammation of the aorta leads to all those alterations of structure in that vessel which are seen in dissections of what are termed aneurisms there—namely, earthy, calcareous, atheromatous, and ulcerous degenerations. If, then, says he, we find similar morbid conditions of the cerebral arteries in apoplexy, we may fairly conclude that they have been produced in the same way as the aortic alterations, namely, by chronic inflammation. The author then proceeds to the facts on which he founds his doctrine.

Fact 1. A female, aged sixty years, experienced, on the 17th September, the precursory symptoms of apoplexy, and was conducted to the Hôtel Dieu on the 18th. She had now paralysis of the right side—great prostration of the intellectual faculties—mouth drawn to the left side—pulse full and regular. An emetic was exhibited; but no improvement resulted. On the contrary, the symptoms indicated cerebral inflammation, and the patient expired on the thirteenth day from the attack.

*Dissection.* The meningeal vessels were gorged with blood. The *right* hemisphere of the brain was firm and healthy. In the middle of the corpus striatum of the *left* side there was an extravasation, or rather an infiltration of blood to the extent of an inch and a half, the surrounding cerebral substance being softened and diffluent as jelly. The lining of the corresponding ventricle was thickened. *The whole of the arteries of the brain were ossified, excepting the smallest twigs.*

*Case* 2. Mad. D. aged 73 years, had experienced some head affection a twelvemonth previously to the night of the 29th January, when she was stricken with apoplexy. She lost her speech, but preserved her intellects to a considerable degree. The *left* side was paralytic, but sensible. She was carried to the Hospital Cochin on the 30th, and died next day.

*Dissection.* The vessels of the dura mater were gorged with blood. The posterior lobe of *the right* hemisphere was a quagmire of extravasated blood, the cerebral substance having been torn up, and a vast depôt collected there. The basilary artery, and indeed all the arteries of the brain, were streaked with white patches of phosphate of lime, and some were completely ossified.

*Case* 3. In this case, which was that of a man 56 years of age, the apoplectic attack proved fatal, and, on dissection, the internal carotid artery was found to have burst within the skull, and just where it begins to branch off to different parts of the brain.

*Case* 4. This was a patient in LA PITIÉ who died of apoplexy, and the basilar artery was found to be aneurismatic. The sac had burst, and blood was extravasated. The coats of the artery were cartilaginous.

*Case* 5. This was very nearly similar, yet the patient was a young female of only 21 years. The basilary artery was aneurismal, and had burst. We need not multiply any more cases to shew that disease of the cerebral vessels is a very common precursor of apoplexy. The only difficulty is to ascertain whether inflammation of the arterial parietes be the cause of these diseased states. The distinguished author of this memoir is decidedly of opinion that chronic phlegmasia is the cause of those calcareous depositions which are seen on vessels, both in the brain and in other parts of the body.

---

